# From Genes to Bioleaching: Unraveling Sulfur Metabolism in *Acidithiobacillus* Genus

**DOI:** 10.3390/genes14091772

**Published:** 2023-09-08

**Authors:** Ana Ibáñez, Sonia Garrido-Chamorro, Juan J. R. Coque, Carlos Barreiro

**Affiliations:** 1 Instituto de Investigación de la Viña y el Vino, Escuela de Ingeniería Agraria, Universidad de León, 24009 León, Spain; aibas@unileon.es (A.I.); jjrubc@unileon.es (J.J.R.C.); 2 Instituto Tecnológico Agrario de Castilla y León (ITACyL), Área de Investigación Agrícola, 47071 Valladolid, Spain; 3 Área de Bioquímica y Biología Molecular, Departamento de Biología Molecular, Universidad de León, 24007 León, Spain; sgarc@unileon.es

**Keywords:** *Acidithiobacillus* genus, sulfur-oxidizing bacteria, sulfur oxidation, Sox complex, SDO, SOR, SQR, HDR-like system, thiosulfate

## Abstract

Sulfur oxidation stands as a pivotal process within the Earth’s sulfur cycle, in which *Acidithiobacillus* species emerge as skillful sulfur-oxidizing bacteria. They are able to efficiently oxidize several reduced inorganic sulfur compounds (RISCs) under extreme conditions for their autotrophic growth. This unique characteristic has made these bacteria a useful tool in bioleaching and biological desulfurization applications. Extensive research has unraveled diverse sulfur metabolism pathways and their corresponding regulatory systems. The metabolic arsenal of the *Acidithiobacillus* genus includes oxidative enzymes such as: (i) elemental sulfur oxidation enzymes, like sulfur dioxygenase (SDO), sulfur oxygenase reductase (SOR), and heterodisulfide reductase (HDR-like system); (ii) enzymes involved in thiosulfate oxidation pathways, including the sulfur oxidation (Sox) system, tetrathionate hydrolase (TetH), and thiosulfate quinone oxidoreductase (TQO); (iii) sulfide oxidation enzymes, like sulfide:quinone oxidoreductase (SQR); and (iv) sulfite oxidation pathways, such as sulfite oxidase (SOX). This review summarizes the current state of the art of sulfur metabolic processes in *Acidithiobacillus* species, which are key players of industrial biomining processes. Furthermore, this manuscript highlights the existing challenges and barriers to further exploring the sulfur metabolism of this peculiar extremophilic genus.

## 1. Introduction

The genus *Acidithiobacillus* recruits a group of mesophilic, aerobic or facultatively anaerobic, obligate acidophilic, chemolithotroph, Gram-negative, rod-shaped bacteria. They are motile by one polar flagellum and are widely distributed in acidic environments where sulfur compounds are observed, like sulfur springs and acid mine drainage waters [1]. In fact, for a long time, *Acidithiobacillus ferrooxidans* was considered the dominant microorganism for metal sulfide bioleaching. However, the progression of genomics knowledge and advancements in molecular bioidentification techniques have spurred the exploration of extreme mineral-leaching environments for novel microorganisms with potential commercial utility. This has led to a more comprehensive understanding of the biodiversity within acidophilic settings. Consequently, new species of archaea and bacteria have garnered increasing attention in recent years. Nonetheless, the presence of *Acidithiobacillus* in all these environments remains unquestionable, and the extensive research conducted over the years boosts its status as a quintessential group of extremophilic acidophilic bacteria in the majority of studies [2].

Under an informal yet effective classification, the genus *Acidithiobacillus* can be categorized into sulfur-oxidizing species (e.g., *Acidithiobacillus thiooxidans*) and iron- and sulfur-oxidizing species (like *A. ferrooxidans*) [3,4]. Additionally, certain species have been reported to utilize H_2_ as an electron donor, including *A. ferrooxidans, Acidithiobacillus ferridurans*, and *Acidithiobacillus caldus* [5]. *Acidithiobacillus thiooxidans* (formerly known as *Thiobacillus thiooxidans*) represents the first species isolated and characterized within the genus, a milestone achieved by Waksman and Joffe in 1921 [6]. At that time, the species that now comprise the *Acidithiobacillus* genus were part of the *Thiobacillus* genus. However, the taxonomic classification within *Thiobacillus* revealed two distinct subgroups, leading to the eventual reclassification of some species into the *Acidithiobacillus* genus in 2000 [7]. Their members belong to the sulfur-oxidizing bacteria (SOB) group, characterized by their ability to catalyze the conversion of elemental sulfur or reduced inorganic sulfur compounds (RISCs) into sulfuric acid, leading to the acidification of their environment, which eases the mobilization of metallic ions [1]. This characteristic has garnered attention in terms of their potential industrial applications, particularly in: (i) the field of biomining for the extraction of valuable minerals (including copper, phosphorus, and even gold), (ii) biohydrometallurgy for the treatment of wastes containing heavy metals (such as sewage sludge, spent household batteries, mine tailings, and printed circuit boards), and (iii) desulfurization of coal and natural gas [8,9,10]. Additionally, *Acidithiobacillus* species act as key players in both sulfur and iron cycles [11].

### 1.1. Taxonomical Classification of the Genus Acidithiobacillus

Initially, *Acidithiobacillus* species were classified within the Betaproteobacteria; members of the Acidithiobacilli class were later reclassified as members of the Gammaproteobacteria. However, with the improvement of genome sequencing and phylogenomic analysis, they were subsequently repositioned as a parallel class to the β-, γ-, and Epsilonproteobacteria, along with *Thermithiobacillus tepidarius.* The class Acidithiobacillia (often referred to as the Acidithiobacilli) contains a single order and two families: the Acidithiobacillaceae and the Thermithiobacillaceae [3].

The genus *Acidithiobacillus* traditionally presented seven species, which can be grouped into two main categories: (i) the iron- and sulfur-oxidizing species, including *A. ferrooxidans*, *Acidithiobacillus ferrivorans*, *A. ferridurans*, and *Acidithiobacillus ferriphilus*; and (ii) the species that solely oxidize sulfur, encompassing *Acidithiobacillus thiooxidans*, *Acidithiobacillus albertensis*, and *A. caldus* [3,4,12]. Nevertheless, recent advancements in taxonomic studies have led to the description of two additional species, namely *Acidithiobacillus sulfuriphilus* [13] (sulfur oxidizer) and *Acidithiobacillus ferrianus* (sulfur/iron oxidizer) [4] (Table 1). Furthermore, genome sequencing efforts have unveiled the presence of three additional new species [3,14]: *Acidithiobacillus marinus* [15], *Acidithiobacillus sulfurivorans* [3], and *Acidithiobacillus montserratensis* [16]. Nonetheless, it is noteworthy to mention that these newly identified species lack a comprehensive morphological and biochemical characterization, as their investigation has been primarily based on molecular analysis through whole-genome sequencing. Thus, the List of Prokaryotic names with Standing in Nomenclature (LPSN) currently recognizes 11 species of *Acidithiobacillus* (https://lpsn.dsmz.de/search?word=Acidithiobacillus, accessed on 12 August 2023) [17].

Indeed, the taxonomic classification of *Acidithiobacillus* members has encountered pervasive challenges brought about by emerging sequencing technologies. This issue has garnered substantial attention, with a growing compendium of recent studies advocating for a reclassification of the genus [3,10,18,19,20,21,22].

### 1.2. Ecological Roles and Physiological Diversity

The ecological significance and widespread distribution of *Acidithiobacillus* spp. are highlighted by their environmental diversity, which includes natural and anthropogenic low-pH environments, such as acidic ponds, lakes, rivers, sulfur springs, acid mine drainage waters, and mining areas found across the globe [10]. These acidophiles are not only of fundamental interest as model organisms for extreme environments but also hold substantial biotechnological and environmental importance [23].

Variability among members of the genus is evident (Table 1). Phenotypically, *Acidithiobacillus* species exhibit a wide range of tolerance for growth temperatures, enduring temperatures exceeding 40 °C in *A. caldus* and as low as 4 °C in *A. ferrivorans*. Furthermore, these microorganisms demonstrate remarkable pH adaptability, displaying substantial variations. Thus, *A. sulfuriphilus* presents an optimal pH of 3, but it can also thrive under neutral conditions with a pH of 7. In contrast, *A. ferrianus* exhibits robust growth even in highly acidic environments, with a pH as low as 1.8 (Table 1) [3]. Thus, the taxa demonstrate a remarkable adaptability, thriving in diverse conditions that span both aerobic and anaerobic environments [5,24,25].

This adaptability is attributed to their inter- and intraspecific divergences, exhibiting both species-to-species and strain-to-strain heterogeneity [3]. These divergent characteristics have facilitated the niche adaptation, endowing *Acidithiobacillus* species with several alternative metabolic pathways for sulfur and iron oxidation. This versatility enables them to exploit diverse energy sources and endure a variety of environmental conditions [26,27,28]. Unraveling these intricate pathways could provide valuable insights into the metabolic versatility of these bacteria [21].

Interestingly, *Acidithiobacillus* species form biofilms, which are essential in the formation of complex communities of microorganisms attached to surfaces. These biofilms play a crucial role in the survival and persistence of *Acidithiobacillus* in extreme environments as they provide protection against environmental stresses and facilitate nutrient acquisition [29].

## 2. Industrial Application of *Acidithiobacillus*

One of the primary industrial applications associated with the members of the *Acidithiobacillus* genus is their utilization in the field of biomining. Biomining entails the utilization of biological systems to facilitate the retrieval and recovery of valuable metals and other industrially significant substances from sulfide minerals, specially from lower-grade sources. The term “bioleaching” is often used interchangeably with biomining, although it more precisely refers to scenarios where the target metal(s) become solubilized during bio-processing [30,31]. On the other hand, “biooxidation” commonly denotes processes in which metal recovery is improved through microbial decomposition of minerals, without the metal itself being solubilized. A prime example of this phenomenon is the recovery of gold from arsenopyrite ores, in which the gold persists within the mineral following biooxidation and is subsequently extracted through the utilization of cyanide [32]. These innovative technologies provide a more sustainable and environmentally friendly alternative to conventional mining practices, which release substantial amounts of CO_2_ emissions [33]. *Acidithiobacillus* species have found significant applications in the advancement of biomining and bioleaching technologies, specifically in the extraction of valuable metals, such as copper (Cu), gold (Au), and phosphorous (P), primarily from low-grade ores and waste materials, although not exclusively.

**Table 1:** Relevant traits of the nine species included in *Acidithiobacillus* genus.

Trait	*A. ferrooxidans*	*A. ferrivorans*	*A. ferriphilus*	*A. ferridurans*	*A. ferrianus*	*A. thiooxidans*	*A. caldus*	*A. sulfuriphilus*	*A. albertensis*
% GC	58.5	56.0	56.5	58.0	58.0	53.0	61.0	61.5	52.5
Cell size (µm)	1.0 × 0.5	2.4 × 0.5	1.0–2.0	1.0–2.0	1.2–2.5	1.0–2.0 × 0.5	1.2–1.9 × 0.7	1.5–2.5 × 0.5	1.0–2.0 × 0.4–0.6
Motility	+/−	+	+	+	+	+	+	+	+
Growth pH (optimum)	1.3–4.5 (2.0–2.5)	1.9–3.4 (2.5)	1.5 (2.0)	1.4–3.0 (2.1)	1.5–3.0 (2.2)	0.5–5.5 (2.0–3.0)	1.0–3.5 (2.0–2.5)	1.8–7.0 (3.0)	2.0–4.5 (3.5–4.0)
Growth T (°C) (optimum)	10–37 (30–35)	4–37 (28–33)	5–33 (30)	10–37 (29)	NR (28–30)	10–37 (28–30)	32–52 (40–45)	15–30 (25–28)	10–40 (25–30)
*Growth on:*									
Sulfur	+	+	+	+	+	+	+	+	+
Thiosulfate	+	+	+	+	+	+	+	+	+
Metal sulfides	+	+	+	+	+/-	+	-	-	-
Ferrous iron	+	+	+	+	+	-	-	-	-
Hydrogen	+	+/-	-	+	+	-	+	-	NR
References	[1,34,35]	[25,34,36]	[24,34]	[34,37,38]	[4]	[1,6,34]	[1,34,39]	[13]	[1,34,40]

Note: “NR” means “Not reported”.

One of the primary interests in the application of *Acidithiobacillus* in the biomining industry lies in its ability to oxidize inorganic sulfur, generating sulfate ions (SO_4_^2−^), resulting in the generation of sulfuric acid (H_2_SO_4_) and its subsequent release into the surrounding medium. This process of environmental acidification provides an economically viable means for various industries to effectively solubilize metals from significant mineral sources. Further, a notable issue in certain leaching and bioleaching processes is the formation of sulfur-rich species on the mineral surface, leading to hindered leaching, a phenomenon known as passivation. Passivation during chalcopyrite bioleaching for copper results in reduced production yields, posing a significant challenge [41]. By promoting the oxidation of these sulfur compounds, *Acidithiobacillus* members, as well as other SOBs, can contribute to maintaining favorable conditions for leaching and preventing passivation, although the effectiveness of *Acidithiobacillus* in mitigating passivation can vary depending on factors such as the specific species, environmental conditions, and the mineral being processed [42,43]. However, *Acidithiobacillus* members exhibit certain limitations that hinder their industrial application, including: (i) long growth cycle, (ii) slow bioleaching rate, and (iii) harsh requirements to grow under controlled conditions [44]. Consequently, the development of these technologies is progressing at a relatively slow pace.

*A. ferrooxidans* originally emerged as the acidophile model microorganism due to its applications in copper biomining [45], and several multi-omic approaches have been carried out in recent years to study its genetic makeup [46,47,48,49]. However, recent studies have underscored the significance of other species, such as *A. thiooxidans*, as noteworthy workhorses in the industrial extraction of a diverse range of metals including Cu, Ni, Zn, and Co from different ores and residues [44,50,51]. Biomining technologies typically operate under extremely acidic conditions with pH levels between 1.5 and 2.5 [52,53], and certain bioleaching approaches (e.g., Monywa (Myanmar)) operate at pH levels below 1.2 [54], which can impact *Acidithobacillus* growth and leaching rates [53,55]. Consequently, certain species exhibit a higher degree of adaptability to this challenging environment than others, which merits further consideration in the context of industrial applications.

However, the lowest pH does not always consistently translate to the best performance [55,56]. The dynamic ecosystem within the bioleaching dump sets the optimal conditions for achieving the highest efficiency. A clear example of this is the case of the mine La Escondida (Chile), where heap bioleaching of low-grade copper ores is carried out without artificial acidification with sulfuric acid. Remarkably, this industrial bioleach heap has been operational for 25 years without the need for prior inoculation, extracting tons of copper. This successful activity has been recently attributed to the presence of sulfur-oxidizing bacteria (SOBs) such as *A. ferrooxidans*, *A. thiooxidans*, and *A. caldus*, as well as different species of *Sulfobacillus* and *Leptospirillum* [45,57]. Thus, the sulfuric acid produced by the biological oxidation of RISCs promotes the solubilization of the Cu, which is then recovered from this acidic solution using physico-chemical technologies, such as solvent extraction and electroplating [45]. This process has yielded an annual average production of over one million metric tons of copper, as reported by the data provider company Statista (https://es.statista.com, accessed on 12 August 2023). Thus, bioleaching accounts for 10% of the copper production worldwide and it is especially important as a technology for ores with a low percentage of mineral that are otherwise uneconomical to extract [45]. The efficiency of this process underscores the importance of understanding and leveraging the natural biological and chemical processes within the bioleaching environment. The role of sulfur-oxidizing bacteria in facilitating the solubilization of copper, for instance, highlights the potential for further optimizing these processes to enhance copper recovery rates.

However, the application of sulfur-oxidizing bacteria (SOBs) extends beyond bioleaching. Their unique capabilities can be harnessed in other industrial applications such as biohydrometallurgy and desulfurization, providing environmentally friendly and cost-effective solutions [58]. In biohydrometallurgy, SOBs play a crucial role in the leaching process. They oxidize the sulfur in metal sulfides, resulting in the release of the metal ions. This biological leaching process is not only more environmentally friendly than traditional methods, but it also allows for the extraction of metals from low-grade ores that would otherwise be considered waste. The use of SOBs in biohydrometallurgy has been successfully demonstrated in several operations worldwide, further validating their potential in this field [59,60]. On the other hand, desulfurization is another area where SOBs can be applied. The burning of fossil fuels releases sulfur dioxide into the atmosphere, contributing to air pollution and acid rain. SOBs can convert this sulfur dioxide into elemental sulfur, which can then be safely removed. This biological desulfurization process is a promising alternative to conventional methods, offering a sustainable solution to the pressing issue of air pollution [58,61].

In conclusion, the versatility of SOBs in various industrial applications underscores their potential in contributing to more sustainable and efficient processes [58]. Their ability to oxidize sulfur not only aids in the extraction of valuable metals from low-grade ores but also helps in mitigating the environmental impact of industrial activities [59]. Moreover, the use of SOBs in desulfurization presents a viable solution to the global challenge of reducing air pollution [58].

Thus, to deliver optimal industrial outcomes, it is crucial to enhance our knowledge at the molecular level of sulfur metabolism in SOBs. Advanced omics technologies, such as metagenomics, transcriptomics, proteomics, and metabolomics, offer unprecedented opportunities to unravel the complex metabolic networks of these microorganisms and their responses to environmental changes. These technologies enable a deeper understanding of sulfur oxidation pathways and the mechanisms underlying the efficiency of SOBs in various industrial processes. By leveraging this knowledge, researchers can optimize the use of SOBs and develop strategies to enhance their activity, stability, and overall performance in industrial applications [61,62]. This manuscript compiles current knowledge on sulfur metabolism in *Acidithiobacillus* to offer an overview of cellular processes, aiding understanding and future research for enhancing industrial processes driven by these mechanisms.

## 3. Elemental Sulfur Metabolism and *Acidithiobacillus*

Despite the presence of iron-oxidizing members within the *Acidithiobacillus* genus, [49] the oxidation of elemental sulfur and diverse reduced sulfur compounds serves as an electron source, enabling *Acidithiobacillus* to drive energy generation through respiratory processes [63]. Proteomic analysis has shown a separation of the iron and sulfur utilization pathways in both iron- and sulfur-oxidizer members, although the two energy-generating pathways can be simultaneously induced depending on the type and the concentration of the available oxidizable substrates [64].

The ability of *Acidithiobacillus* species to oxidize sulfur was first detected in *A. thiooxidans* in 1959 by in vitro assays [65]. Since then, the identification of sulfur-oxidizing enzymes has remained a central focus of the field. The sulfur cycle is a vital biogeochemical process that affects ecosystem functioning, atmospheric chemistry, and nutrient availability. Sulfur can exist in different oxidation states ranging from −2 to +6, leading to the formation of a variety of reduced/oxidized inorganic sulfur compounds, including thiosulfate (S_2_O_3_^2−^), sulfite (SO_3_^2−^), sulfate (SO_4_^2−^), tetrathionate (S_4_O_6_^2−^), and elemental sulfur (S^0^), among others [66]. This transformation of sulfur compounds is a key step in the cycle, and it is mediated by a diverse array of microorganisms. Among these, *Acidithiobacillus* stands out due to its acidophilic nature, contributing to the sulfur cycle in acidic environments [66].

In an *Acidithiobacillus* cell, the electrons derived from the reduction of sulfur are initially transferred to oxidized glutathione (GSSH) and then proceed through a series of sulfur transporters before ultimately entering the quinone pool. From there, the electrons can follow two possible routes: (i) they may directly and sequentially pass to the terminal enzyme complex to generate reducing power, or (ii) they can be transmitted to the NADH complex for the same purpose.

The adherence of bacterial cells to sulfur is a crucial step in the sulfur oxidation process in *Acidithiobacillus* species. When utilizing sulfur as a substrate, *A. ferrooxidans* exhibits an increased synthesis of fatty acids and lipid compounds in extracellular polymeric substances (EPS), which enhance bacterial adhesion to sulfur particles through hydrophobic interactions [49,67]. Similarly, in *A. thiooxidans*, phospholipids production has been reported as responsible for wetting elemental sulfur, an essential requirement for bacterial growth [68]. Thus, following cell adhesion to sulfur particles (including the orthombic α-S_8_, the main elemental sulfur form in nature), elemental sulfur is activated upon contact with a thiol group (RSH), such as that found in the outer-membrane proteins of the *Acidithiobacillus* genus. This activation process may induce the opening of the α-S8 ring, leading to the formation of linear polysulfide, enabling its entry into the bacterial cell and initiating its metabolism [67,69].

**Figure 1:**
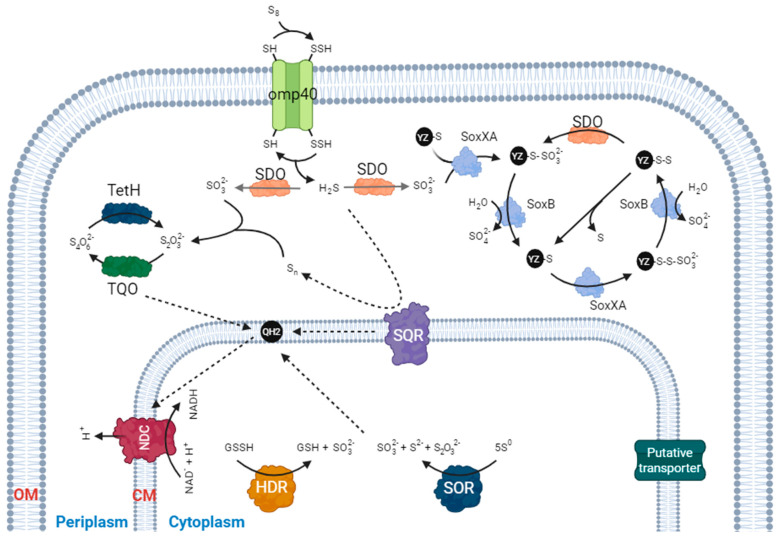
Model of sulfur oxidation metabolism in *Acidithiobacillus* species, involving both the periplasm and the cytoplasm, within a complex metabolic pathway that encompasses numerous enzymes and transporters. SDO, sulfur dioxygenase; TQO, thiosulfate quinone oxidoreductase; SQR, sulfide:quinone oxidoreductase; QH2, quinol pool; NDC, NADH dehydrogenase complex; HDR, HDR-like complex; SOR, sulfur oxygenase reductase; OM, outer membrane; CM: cytoplasmic membrane. Created by BioRender.com.

Nevertheless, since unraveling these peculiar pathways could provide valuable insights into the metabolic versatility of these bacteria and how they adapt to different environments [70], a comprehensive understanding of this intricate metabolic pathway needs a deep analysis of the proteins and processes involved (Figure 1). Following sections describe the principal enzymatic activities currently known in *Acidithiobacillus* for elemental sulfur metabolism, including those present in the periplasm (e.g., sulfur dioxygenase) and in the cytoplasm (such as sulfur oxygenase reductase).

### 3.1. Sulfur Dioxygenase (SDO)

Sulfur oxidation in the *Acidithiobacillus* genus is initiated by the enzyme sulfur dioxygenase (SDO; EC 1.13.11.18) [36,71], which is one of the earliest reported enzymes involved in the process. It was first isolated in 1987 from *A. ferrooxidans* and is composed by 21 and 26 kDa subunits in *A. thiooxidans* or two 23 kDa subunits in *A. ferrooxidans* [72,73,74]. This periplasmic enzyme (Figure 1) functions as a sulfur:ferric ion oxidoreductase and is able to catalyze the oxidation of S^0^ to sulfite [49,63]. However, the genes responsible for the biosynthesis of this enzyme (also known as *sdo* genes) were identified almost thirty years later by Wang and co-workers [75]. Subsequent studies have led to the proposal of two homologs in mitochondria and heterotrophic bacteria: ETHE1 and PDO (persulfide dioxygenase), respectively, both of which have been extensively investigated [76,77,78,79,80]. The ETHE1/PDO complex cooperates with the sulfide:quinone oxidoreductase (SQR) to oxidize H_2_S, thereby diminishing the toxic impact of H_2_S on cellular processes [77,80]. However, the specific role of SDO in *Acidithiobacillus* species remains unknown. It has been postulated that sulfur may be adsorbed onto the cell surface by extracellular polymeric substances [49] and, as a result, it is transported to the periplasmic space following activation by a thiol-containing outer-membrane protein (OMP) to form persulfide sulfane sulfur (Figure 1). Subsequently, SDO might function as the primary enzyme for sulfur oxidation, leading to the production of sulfite [81]. Although not yet conclusively demonstrated, there are indications that the OMP responsible for intracellular sulfur uptake is likely the Omp40 (AFE2741) protein. Omp40 is a 40 kDa protein that forms an oligomeric structure of 120 kDa, associated with the adhesion to solid sulfur substrates and regarded as an adaptation to hinder the unrestricted movement of protons across the outer membrane in *A. ferrooxidans* [82]. Its expression is enhanced during the growth of *A. ferrooxidans* on sulfur, but not in the exclusive presence of iron, leading to the proposal that Omp40 might function as a potential sulfur transporter [83].

Additionally, recent research has revealed the presence of two or even three copies of SDO paralogs in certain strains of *Acidithiobacillus* [34], contributing to the intricacy of comprehending sulfur metabolism in these organisms. Such is the case for *A. caldus* MTH-04, where two homologs have been identified, named SDO1 (A5904_0421) and SDO2 (A5904_07909) [63]. Consequently, a detailed analysis of the SDO genes in *Acidithiobacillus* has shown that the homologous found from different species can be classified into four principal subgroups: ETHE1, Blh, SdoS, and SdoA. Other examples are *A. thiooxidans* A01 (SdoS; WP_024895058.1 and SdoA; WP_024893175.1), *A. thiooxidans* ZBY (SdoS; WP-024895058.1 and SdoA; WP_024893175.1), and *A. ferridurans* JCM_18981 (SdoS; BBF65856.1 and ETHE1; BBF64918.1) [34]. Indeed, the existence of various SDO subgroups suggests their potential involvement in distinct pathways for elemental sulfur oxidation. The ETHE1 subgroup appears to be associated with the H_2_S pathway, whereas the SdoS subgroup is likely connected to the S_4_O_6_^2−^ decomposition pathway [63]. This diversity in SDO subgroups implies a sophisticated regulation and coordination of sulfur metabolism in *Acidithiobacillus* species.

### 3.2. Sulfur Oxygenase Reductase (SOR)

Sulfur oxygenase reductase (SOR) is another well-known elemental sulfur-oxidizing enzyme. It catalyzes the oxygen-dependent disproportionation of elemental sulfur, a process in which sulfur molecules undergo a chemical transformation. This results in the production of several compounds, namely sulfite (SO_3_), thiosulfate (S_2_O_3_), and sulfide (H_2_S) (Figure 1). This enzymatic reaction plays a crucial role in the sulfur metabolism of *Acidithiobacillus* and contributes to the conversion of elemental sulfur into various sulfur compounds, which have significant ecological and biochemical implications [34].

First considered an “archaeal like” enzyme, it is also encoded in the genome of some acidophilic leaching bacteria such as some *Acidithiobacillus* species [84]. Once again, the intricacies surrounding the mechanistic action of SOR surpass our current understanding. While the activity of SOR in *A. caldus* SM-1 was initially reported by Chen and co-workers in 2007 [85], subsequent investigations revealed the absence of the corresponding gene in the strain’s genome [86]. Finally, the observed enzymatic activity was attributed to sample contamination by *Sulfobacillus* [84]. However, despite this aforementioned discovery, genes encoding SOR have been identified in some, but not all, strains of *A. thiooxidans, A. ferrooxidans, A. ferrivorans, A. caldus*, and *A. albertensis* [36,43,87,88,89]. Phylogenetic analysis strongly suggests that the identified SORs in these *Acidithiobacillus* strains were likely acquired through horizontal gene transfer from sulfur-oxidizing archaea [34]. In fact, several analyses indicate that SOR activity is secondary, rather than essential, for cytoplasmic elemental sulfur oxidation in these sulfur-oxidizing bacteria [34,84,86,88].

### 3.3. Heterodisulfide Reductase (HDR)-like System

The next step in the sulfur metabolism is postulated to occur in the cytoplasm by a heterodisulfide reductase (HDR)-like system, which serves as an elemental sulfur oxidation enzyme in *Acidithiobacillus* and other sulfur-oxidizing bacteria and archaea [36,88,90,91,92], since transcriptomic analyses in *A. thiooxidans* have reported an increase in its expression levels in the presence of elemental sulfur compared to other sulfur sources such as thiosulfate [93].

This complex is proposed to be involved in the oxidation of disulfide intermediates, particularly sulfane sulfur species like GSSH (oxidized glutathione) or other sulfur carriers, finally converting them into sulfite. Nevertheless, concrete biochemical evidence in *Acidithiobacillus* supporting the function of HDR-like systems remains diffuse. Recently, indirect analyses have provided evidence of the role of the HDR-like complex in the oxidation of thiosulfate to sulfite in the α-proteobacteria *Hyphomicrobium denitrificans* [94], with the lipoate-binding protein LbpA being essential in this process [95]. Additionally, *hdr*-like genes are consistently found in conjunction with genes encoding the TusA protein and other rhodanase homologs [91]. TusA and DsrE are sulfur carrier proteins that exist in several sulfur-oxidizing bacteria and archaea [90,91]. *rhd*-*tus*A-*dsr*E genes have been reported either individually or as part of larger gene clusters in *Acidithiobacillus* species, including *A. caldus* and *A. ferrooxidans*, among others [34,90]. Thus, a sulfur oxidation pathway has been proposed, involving the HDR-like complex responsible for the oxidation of sulfane sulfur to sulfite developed by the carrier TusA. The electron transfer in this reaction may be facilitated by LbpA, leading to the generation of NADH [95]. The confirmation of the sulfur-oxidizing ability of the HDR-like complex and its involvement in the sulfur-metabolizing process demonstrated in *H. denitrificans* [94], *Metallosphaera cuprina*, and *Allochromatium vinosum* [90,91] holds the potential to provide valuable insights into the elemental sulfur oxidation mechanisms operating within the cytoplasm of *Acidithiobacillus* species. Consequently, further research on this complex is warranted.

In these aforementioned species, it has been also reported that inorganic sulfur compounds are successively transferred by the rhodanase (Rhd), as well as the carriers DsrE and TusA, producing sulfane sulfur at the Cys18 of TusA [90,91]. Rhd, which belongs to the sulfur transferase family found in organisms from all three domains of life, is known to participate in various cellular processes [34]. This enzyme cleaves the S–S bond in thiosulfate, producing sulfur and sulfite. Interestingly, Rhd has been purified from crude extracts of *A. ferrooxidans, A. caldus*, and *A. thiooxidans* [96,97], and its gene sequences have been identified in the complete genomes of *Acidithiobacillus* species [45,89]. However, its precise involvement in sulfur metabolism within *Acidithiobacillus* remains to be fully elucidated, since transcriptomic analyses have indicated low expression of the *rhd* gene in *A. thiooxidans* during both thiosulfate and sulfur growth conditions [93].

On the other hand, TusA plays a central role in sulfur movements within the cytoplasm of sulfur-oxidizing prokaryotes [91] and might deliver sulfane sulfur to the HDR-like system [95], thus acting as a crucial link between sulfur transferal and the HDR-like complex. Given the presence of these genes in certain *Acidithiobacillus* strains, it is plausible that similar pathways might operate in the cytoplasm of these sulfur-oxidizing bacteria. Consequently, conducting proteomic characterization and in vivo functional studies of these genes would be of great interest to confirm the functionality of the proposed process and shed light on the sulfur metabolism in *Acidithiobacillus*.

The coexistence of SDO, SOR, and the HDR-like complex in the *Acidithiobacillus* genus highlights the diversity and intricacy of elemental sulfur oxidation within these acidophilic bacteria. Notably, a triple *sor*-*sdo*1-*sdo*2 mutant of *A. caldus* MTH-04 displayed heightened elemental sulfur oxidation activity, suggesting the presence of as-yet-undetermined elemental sulfur oxidation enzymes in *Acidithiobacillus* species [63].

## 4. Beyond Elemental Sulfur: Other Essential Pathways in Sulfur Metabolism

The sulfur metabolism extends beyond the processes elucidated thus far. Sulfur-oxidizing bacteria exhibit a remarkable capacity to utilize alternative sulfur sources, such as thiosulfate or metal sulfides, in addition to elemental sulfur [1]. Furthermore, the metabolism of sulfur compounds often gives rise to various intermediate products, resulting in the existence of multiple interconnected metabolic pathways within these microorganisms. The diversity of these metabolic pathways underscores the adaptability and versatility of sulfur-oxidizing bacteria, enabling them to thrive in diverse environmental conditions and exploit a wide range of sulfur compounds as energy sources. Hereafter, the metabolism of alternative sulfur sources is detailed as far as it is known.

### 4.1. Thiosulfate

Thiosulfate (S_2_O_3_^2−^) assumes a main role in the biogeochemical sulfur cycle, serving as a common substrate for *Acidithiobacillus* species and a key metabolic intermediate oxidized by almost all sulfur-oxidizing microorganisms [34]. For this purpose, all *Acidithiobacillus* species harbor an elaborate thiosulfate-oxidizing multi-enzyme system, which efficiently transforms thiosulfate into other forms of sulfur substrates. Consequently, thiosulfate can be oxidized through either the Sox (sulfur oxidation) system or the TQO (thiosulfate:quinone oxidoreductase) pathway (Figure 1).

**Figure 2:**
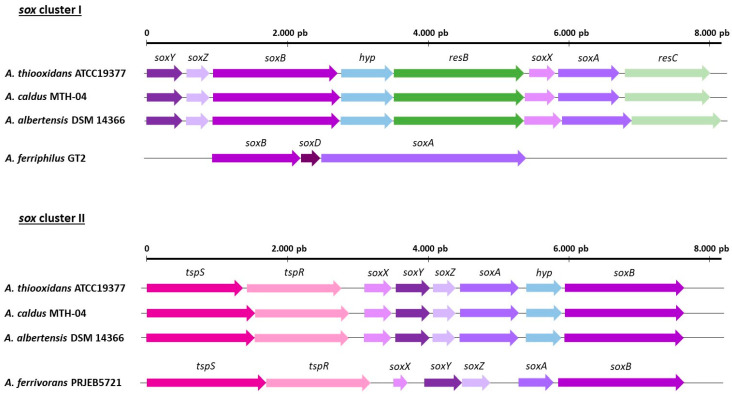
The sox clusters I and II in different *Acidithiobacillus* strains: *A. caldus* MTH-04, *A. thiooxidans* ATCC 19377, *A. albertensis* DSM 14366, *A. ferrivorans* PRJEB5721, and *A. ferriphilus* GT2.

The Sox system, initially discovered in the α-proteobacterium *Paracoccus pantotrophus*, stands as one of the extensively investigated sulfur oxidation systems [98,99]. This periplasmic multi-enzyme complex (Figure 1) exhibits a broad distribution among both photo- and chemo-lithotrophic sulfur-oxidizing prokaryotes [34], including several species of *Acidithiobacillus*, such as *A. ferrivorans*, *A. caldus*, *A. thiooxidans*, and *A. ferriphilus* [36]. Primarily situated in the periplasm, the Sox system consists of SoxXA, SoxYZ, SoxB, and Sox(CD)_2_ in most sulfur-oxidizing bacteria. Nonetheless, in the majority of *Acidithiobacillus* members, the Sox cluster is found to be truncated. Consequently, while the Sox system is present across multiple *Acidithiobacillus* species, the specific constituents and reactions it encompasses vary among them. In contrast to non-iron-oxidizing species such as *A. thiooxidans, A. caldus,* and *A. albertensis*, which possess a cluster of predicted essential genes (specifically *soxABXYZ*), this conservation is not observed in the analyzed iron-oxidizing species (Figure 2). Furthermore, it is customary to encounter dual Sox clusters in non-iron-oxidizing species, displaying a distinct gene arrangement for the sox genes. Moreover, within *sox* cluster I, *resBC* genes are also located, both intricately linked with the maturation of cytochrome C in *A. ferrooxidans* [100]. Indeed, an interruption of the *resB* gene in *A. ferrooxidans* ATCC 19859 yielded a mutant incapable of iron oxidation, while retaining sulfur oxidation proficiency [101]. Nevertheless, within *sox* cluster II, the conspicuous presence of *tspSR* genes is discerned, comprising a σ^54^-dependent two-component system that potentially interfaces with the signal transduction and transcriptional regulatory milieu of the Sox system in *A. caldus* and *A. thiooxidans* [34,102].

Interestingly, transcriptomic analysis conducted in *A. thiooxidans* has unveiled a dynamic modulation of the relative expression of the two *sox* clusters, dependent on the environmental pH and the sulfur source. Notably, these clusters exhibit elevated expression levels during thiosulfate-based growth, as opposed to elemental sulfur utilization [93].

**Figure 3:**
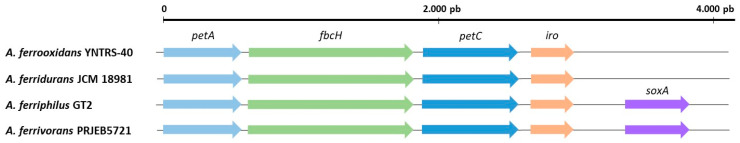
The iron oxidation cluster in different *Acidithiobacillus* strains: *A. ferrooxidans* YNTRS-40, *A. ferridurans* JCM 98981, *A. ferrivorans* PRJEB5721, and *A. ferriphilus* GT2.

On the other hand, certain iron-oxidizing species, such as *A. ferrivorans,* exhibit a single copy of the *sox* cluster, whereas others, like *A. ferriphilus,* present a modified and truncated version of the cluster (genes *sox*ABD). In the cases of *A. ferrooxidans* and *A. ferridurans*, no discernible presence of the cluster has been identified. Lastly, in some strains of *A. ferriphilus* and *A. ferrivorans*, the gene *soxA* has been observed to be clustered together with iron oxidation genes (*iro*) and cytochrome C (*petAC*) genes (Figure 3). On the one hand, both pet genes are responsible for encoding cytochrome C, an integral component of the respiratory chain within iron/sulfur-oxidizing *Acidithiobacillus* species [3]. Conversely, the *fbcH* gene has been associated with cytochromes that are believed to play roles in Fe redox reactions [103].

These contrasting genetic profiles imply that iron-oxidizing species have likely undergone distinct evolutionary pathways, resulting in alternative mechanisms to facilitate sulfur oxidation processes [87]. Partial presence of the sox gene cluster has been reported in strains of early diverging iron/sulfur-oxidizing species (such as *A. ferrianus, A. ferriphilus,* and *A. ferrivorans*), although the absence of soxXA cytochromes in their genomes may indicate a gradual degeneration of the *sox* system in iron/sulfur oxidizers. This theory finds support in the absence of remnants of the cluster in the late-diverging iron/sulfur-oxidizing species (*A. ferrooxidans* and *A. ferridurans*) [3].

In *P. pantotrophus*, the Sox system consists of four components: SoxXA, SoxYZ, SoxB, and Sox(CD)_2_. First, SoxXA catalyzes the reaction between the sulfane sulfur of thiosulfate (S_2_O_3_^2−^) and the SoxY-cysteine-sulfhydryl group of the SoxYZ complex, forming a cysteine S-thiosulfonate derivative (SoxYZ-S-S-SO_3_^−^). Then, SoxB hydrolyzes sulfate (SO_4_^2−^) from the thiocysteine-S-sulfate residue (SoxYZ-S-S-SO_3_^−^). Third, Sox(CD)_2_ may oxidize the outer sulfur atom of S-thiocysteine, producing SoxYZ-cysteine-S-sulfate (SoxYZ-S-SO_3_^−^). Finally, sulfate is hydrolyzed and removed by SoxB from SoxYZ-S-SO_3_^−^, and SoxYZ is regenerated [88]. In a similar manner, the enzymes located in the genome of *Acidithiobacillus* species are expected to assume these roles and carry out a comparable sequence of reactions.

**Figure 4:**
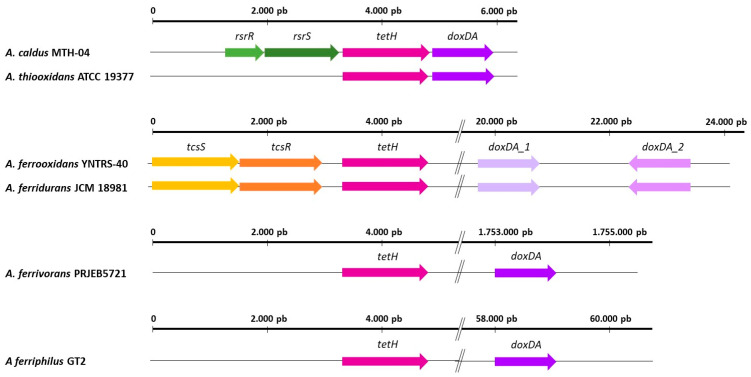
Arrangement of genes of S4I pathway in different *Acidithiobacillus* strains: *A. caldus* MTH-04, *A. thiooxidans* ATCC 19377, *A. ferrooxidans* YNTRS-40, *A. ferridurans* JCM 98981, *A. ferrivorans* PRJEB5721, and *A. ferriphilus* GT2. Double lines indicate a genome gap. Note that empty spaces around the described genes are not annotated genome regions.

TQO (thiosulfate:quinone oxidoreductase) was initially identified in the archaea *Acidiaus ambivalens* and consists of two 28 kDa DoxA and two 16 kDa DoxD subunits [104]. This enzyme is responsible for the oxidation of thiosulfate to tetrathionate (Figure 1). Phylogenetic analysis of some *Acidithiobacillus* species revealed that the subunits DoxD and DoxA have fused into a single protein [105] (Figure 4). To date, the catalytic mechanism of TQO in *Acidithiobacillus* remains uncertain, and additional experimental investigations are required to confirm its functional role in sulfur oxidation. The subsequent hydrolysis of tetrathionate to thiosulfate and other products is facilitated by the enzyme TetH. The hydrolytic activity of tetrathionate hydrolase has been extensively studied in *Acidithiobacillus*, encompassing investigations into its enzymatic properties, protein localization, and functional role in the sulfur metabolic network [36,88,89]. In the genomes of *A. caldus* and *A. thiooxidans,* the *tetH* and *dox*DA genes are found in a cluster (Figure 4), whereas they are located separately in the genomes of the iron-oxidizing strains *A. ferrooxidans*, *A. ferridurans, A. ferrivorans*, and *A. ferriphilus*. Moreover, the transcription of *tetH* and *doxDA* is significantly influenced by different sulfur substrates present in growth media, suggesting that *Acidithiobacillus* species can modulate the pathway at the transcriptional level in response to different sulfur metabolites present in the environment. Notably, studies have revealed that *tetH* expression is subject to varying degrees of upregulation in the presence of tetrathionate, thiosulfate, and pyrite in *A. caldus* [106], whereas transcriptomic analysis in *A. thiooxidans* showed an elevation in expression levels during stationary growth on thiosulfate, implying a distinct functional role of this protein in utilizing thiosulfate as a growth source [93]. Additionally, a *tetH* knockout mutant demonstrated a drop in both *A. ferrooxidans* and *A. caldus* growth using tetrathionate as the sole energy source [107,108]. Lastly, TetH enzymes purified from *A. thiooxidans, A. ferrooxidans*, and *A. caldus* exhibit homodimeric structures, and their optimal enzymatic activities are detected under acidic conditions, typically at pH levels of 3.0–4.0 [34,109,110].

### 4.2. Sulfide Oxidation

Sulfide (S^2−^) is an important sulfur substrate and metabolic intermediate in elemental sulfur oxidation in *Acidithiobacillus*. As previously stated, sulfur gains access to the cell periplasm through a transmembrane protein, OMP, employing a thiol residue. Subsequently, the production of hydrogen sulfide (H_2_S) takes place, which is susceptible to oxidation by the membrane-bound enzyme sulfide:quinone oxidoreductase (SQR), leading to the formation of zero-valent sulfur. This oxidation process also results in the generation of electrons, which contribute to the electron flow within the membrane-associated quinone pool [111,112].

In 2010, the crystal structure of *A. ferrooxidans* SQR was determined by Cherney and co-workers [113]. The active site of this enzyme encompasses two cysteine residues (Cys160 and Cys356) that play a critical role in electron transfer to the flavin adenine dinucleotide (FAD) cofactor. Additionally, a third cysteine residue (Cys128) and two histidine residues (His132 and His198) are deemed essential for its enzymatic function. It has been proposed that when a sulfide ion interacts with the sulfur atom of Cys356, two electrons are transferred to the FAD moiety, facilitating the oxidation reaction within the catalytic site [113,114].

Nevertheless, despite the proposition of various putative *sqr* genes in other *Acidithiobacillus* species [15,115], their precise roles in sulfur oxidation remain unclear. Furthermore, the existence of alternative pathways in the remaining species, which have not yet been described, cannot be ruled out.

### 4.3. Sulfite Oxidation

Sulfite (SO_3_^2−^) is characterized as metastable and relatively short-lived within mine waste environments. One plausible scenario is that sulfite swiftly undergoes non-enzymatic oxidation to form sulfate, thiosulfate, or glutathione S-sulfonate in the presence of Fe(III) or sulfur [116]. Furthermore, it is a potent cellular toxin that must be rapidly detoxified to prevent cellular damage.

The process of sulfite oxidation is evident in numerous bacteria, including *Acidithiobacillus*, and can occur in both the periplasm and the cytoplasm, mediated by a suite of specialized enzymes [21,36,70,71]. While proteins catalyzing this reaction have been isolated from different *A. ferrooxidans* strains, genes encoding well-known periplasmic enzymes engaged in the direct oxidation of sulfite during dissimilatory sulfur metabolism (*sorAB* or *soxCD*) have not been identified within the *A. ferrooxidans* genome [116].

Sulfite is postulated to be generated in the cytoplasm through heterodisulfide reductase, subsequently being converted to adenosine-5′-phosphosulfate (APS) by the extensively characterized APS reductase complex encoded by *aprBA*. However, the genome lacks identifiable candidates significantly resembling *aprBA*. Nonetheless, a predicted *sat* gene (AFE_0539) is present which, in other microorganisms, encodes an ATP sulfurylase involved in the second step of this pathway. *A. ferrooxidans* Sat shares 44% identity and 60% similarity with both domains of the bifunctional SAT/APS kinase from *Aquifex aeolicus*, which catalyzes the synthesis of ATP and sulfate from APS and pyrophosphate [116].

On the other hand, periplasmic sulfite can be converted to sulfate via the action of sulfite oxidase (SOX) (Figure 1), a molybdenum-containing enzyme that catalyzes the two-electron oxidation of sulfite to sulfate. This reaction is a key step in the sulfur oxidation pathway and plays a crucial role in the energy metabolism of *Acidithiobacillus* and other sulfur-oxidizing bacteria [21,36,70,71]. Alternatively, sulfite can be converted to thiosulfate by thiosulfate/3-mercaptopyruvate sulfur transferase. This enzyme catalyzes the transfer of a sulfur atom from sulfite to another sulfur compound, resulting in thiosulfate formation. Thiosulfate can then be further oxidized to sulfate by the enzyme thiosulfate:quinone oxidoreductase (TQO), contributing to the metabolic versatility of *Acidithiobacillus* [36,90,105,110]. In addition to these pathways, sulfite can also be conjugated to glutathione to form glutathione S-sulfonate (GSSO_3_H) in a reaction catalyzed by glutathione-S-transferase. This reaction serves as a detoxification mechanism, allowing the cell to safely sequester sulfite and prevent its toxic effects [61,65,117].

## 5. Genetic and Molecular Aspects of *Acidithiobacillus*

This overview of sulfur metabolism in *Acidithiobacillus* presents the main partners of the sulfur metabolic pathways, but it also highlights the extensive unknown aspects surrounding these metabolic pathways. It is well established that genomic sequencing has significantly contributed to the molecular understanding of a plethora of microorganisms, including their basal metabolism. However, when it comes to in-depth analyses of these genes, particularly for applications in synthetic biology, specialized tools and techniques are essential.

To date, only a handful of vectors enable the transformation (such as pBBR1MCS-6, pMSD1, pBBR-tac-Sm, and pMSD2) [118] or conjugation (e.g., pJRD215 and pSIM-PLE19hsdM::Ω-Cm) [119] of a couple of *Acidithiobacillus* species (*A. caldus*, *A. thiooxidans*). All of these vectors rely on the streptomycin resistance gene as the selection marker for transformants, which is a critical attribute given the limited availability of antibiotics suitable for selection in extremely acidophilic environments [118,120,121]. However, the efficacy of these plasmids in other *Acidithiobacillus* species has to be confirmed.

More recently, the emergence of gene-editing systems centered around CRISPR has led to the development of pAFi and pAF systems (CRISPR-dCas9 and CRISPR-Cas9-based, respectively). These systems offer valuable tools for genetic engineering in *A. ferridurans* [122], although their effectiveness in other species remains unexplored.

Consequently, the scarcity of genetic editing techniques enabling knockout studies poses a challenge in comprehensively investigating the metabolic pathways outlined throughout this manuscript.

Additionally, the application of genomic sequencing to *Acidithiobacillus* species presents peculiar challenges [123]. The assembly and annotation of genomic sequences in these cases can be particularly demanding due to the intricacy of their genomes. A notable limitation stems from the scarcity of complete and thoroughly annotated genomic sequences for most *Acidithiobacillus* species. Despite the sequencing and publication of numerous genomes, their annotation remains incomplete or inconsistent [124], which hampers comparisons between species and identification of key genes and metabolic pathways [93]. Furthermore, a significant proportion of genes within these genomes have unknown functions, further complicating the understanding of these organisms [3,125] (Table 2). Certainly, *A. ferrooxidans* stands as a conspicuous exemplar. It has garnered extensive scrutiny owing to its remarkable industrial applications. Nonetheless, out of the 64 genomes attributed to this species and deposited in the NCBI database, merely 5 have attained complete assembly, signifying the continuing challenges in achieving comprehensive genomic characterization. Far behind, *A. thiooxidans* and *A. caldus* have 26 and 24 genomes, respectively, with only 1 and 4 of them fully assembled, respectively. Meanwhile, species such as *A. ferrianus, A. sulfuriphilus*, and *A. albertensis* are left behind, as no complete assembled genome has been made available for them. Moreover, it is noteworthy that approximately 40% of the genomes from the currently described species consist of hypothetical proteins, as annotated by the RAST annotation service (Table 2). Further efforts in genomic research will boost the understanding of biology and functional attributes of these *Acidithiobacillus* spp.

The traditional challenges of genetic characterization cannot be overlooked. Thus, the translation of findings from genomic studies into practical applications is another significant hurdle. Genomic data can offer valuable insights into the metabolic capabilities and environmental adaptations of *Acidithiobacillus* species, but harnessing this knowledge for biotechnological applications or environmental management requires a deep comprehension of the functional implications of the genomic data [126].

These gaps in genomic information contribute to the limited outcomes of omics analyses conducted with *Acidithiobacillus*, encompassing studies involving sulfur metabolism [64] or other investigations such as stress assessment [22,127,128] and molecular processes during biomining operations [129]. In most of the transcriptomic and proteomic analyses, a significant percentage of genes and proteins remain unidentified, which hampers the omics analyses as has recently been described by Ibañez and co-workers [22]. Consequently, while these analyses confirm the presence of numerous genes involved in these molecular processes, attributing specific roles to these genes often proves challenging. For instance, a proteomic analysis of the periplasm of *A. ferrooxidans* ATCC 23270 reveals over 200 proteins associated with thiosulfate, elemental sulfur, and ferrous iron metabolism. Nevertheless, approximately 34% of these proteins remain uncharacterized, limiting the ability to establish a definitive working model for sulfur oxidation in *A. ferrooxidans* [67].

## 6. Conclusions

Sulfur oxidation in chemoautotrophic *Acidithiobacillus* constitutes a vital aspect of microbial sulfur metabolism within the development of biomining and bioremediation technologies. In that regard, significant progress has been achieved in the understanding of *Acidithiobacillus* sulfur oxidation. Sulfur metabolism in *Acidithiobacillus* spp. encompasses multiple semistable sulfur oxidation intermediate (SOI) compounds and a range of sulfur-oxidizing pathways and enzymes located in different cellular compartments, highlighting the complexity and diversity of the metabolism in these acidophilic autotrophic bacteria.

However, despite these advances, detailed and comprehensive investigations into gene functions and enzymatic properties involved in sulfur oxidation are still scarce, leading to certain aspects of sulfur metabolism in *Acidithiobacillus* remaining ambiguous or unanswered. Most of these SOI compounds are either not comprehensively constrained and/or lack readily available analytical methods for their characterization. For instance, the challenges in measuring polythionates and other higher-oxidation-state sulfur compounds have impeded the delineation of their roles in the chain of reactions. Likewise, the existence of multiple sulfur-oxidizing genes and pathways, as well as their different roles in sulfur oxidation, the enzymatic properties of certain proteins, and the structural analysis and catalytic mechanisms of certain enzymes require further clarification.

To address these knowledge gaps, the integration of different omics technologies at the DNA, RNA, and protein levels can aid in the discovery of new sulfur-oxidizing proteins and enhance our comprehension of sulfur metabolic networks in different *Acidithiobacillus* strains. Furthermore, the development of novel methods or techniques, such as visualization of protein localization, as well as in vivo and in vitro sulfur metabolite detection, will facilitate the investigation of enzymatic function and catalytic processes in *Acidithiobacillus* species.

## Figures and Tables

**Table 2:** Data summary of the genomes of *Acidithiobacillus* species available in the NCBI Database. The genomes used as a reference in the analysis are those considered by the database as “NCBI reference genomes” (the best assembled deposited biotype is selected as reference one). These genomes are always one biotype from the indicated strains (2nd row).

	*A. ferrooxidans*	*A. ferrivorans*	*A. ferriphilus*	*A. ferridurans*	*A. ferrianus*	*A. thiooxidans*	*A. caldus*	*A. sulfuriphilus*	*A. albertensis*
Reference genome	ASM1346280v1	NEW_PRJEB5721	ASM2084402v1	ASM396665v1	ASM1037809v1	ASM966247v1	ASM869422v1	ASM372122v1	ASM193165v1
Strain	YNTRS-40	PRJEB5721	GT2	JCM 18981	MG	ATCC 19377	MTH-04	CJ-2	DSM 14366
Deposited genomes	64	12	12	9	1	26	24	1	2
Completed genomes	5	3	2	1	0	1	4	0	0
Total genes	3542	3781	2633	3173	3467	3707	2995	3083	3909
Hypothetical proteins	38.18%	43.67%	34.26%	36.53%	39.72%	45.75%	41.17%	38.11%	43.34%

Note: A genome is considered complete, according to the NCBI definition, “when all chromosomes are gapless and contain no runs of 10 or more ambiguous bases (Ns). Additionally, there should be no unplaced or unlocalized scaffolds, and all the expected chromosomes must be present. Plasmids may or may not be included in the assembly, but if present, their sequences should be gapless”. Total genes and hypothetical proteins were determined after RAST server annotation of type strains for each species.

## Data Availability

Not applicable.
